# The Production and Durability of Superhydrophobic Foamed Concrete

**DOI:** 10.3390/ma18030663

**Published:** 2025-02-02

**Authors:** Juntao Mao, Yi Xu, Xuan Kang, Songru Tong, Hongqiang Chu, Linhua Jiang

**Affiliations:** 1 College of Material Science and Engineering, Hohai University, Nanjing 211100, China; maojt1998@163.com (J.M.); tomyoo86@163.com (X.K.); tsr106792@163.com (S.T.); 2 College of Civil and Transportation Engineering, Hohai University, Nanjing 211100, China; chq782009@126.com (H.C.); lhjiang@hhu.edu.cn (L.J.)

**Keywords:** superhydrophobic, foamed concrete, durability, binary synergistic, 3D surface morphology

## Abstract

The durability problem caused by the high-water absorption of foamed concrete restricts its further development and application. This study aimed to improve the durability of foamed concrete by transforming its performance from hydrophilic to superhydrophobic. Firstly, polydimethylsiloxane-modified superhydrophobic bulk foamed concrete was produced through physical foaming. Then, multiple durability tests, like mechanical wear, acid–alkali–saline resistance, ultraviolet aging, and extreme temperatures resistance tests, were carried out to assess its performance. Finally, the mechanism of superhydrophobicity also was studied. The results indicated that the volumetric and capillary water absorption of the superhydrophobic foamed concrete decreased by 72.4% and 92.6%, respectively, compared to ordinary foamed concrete. The dry densities of ordinary foamed concrete and superhydrophobic foamed concrete were 720 kg/m^3^ and 850 kg/m^3^, respectively. Superhydrophobic foamed concrete exhibited excellent wear resistance and resistance to ultraviolet aging. The contact angles after 10 m polishing and 168 h of ultraviolet irradiation were 152.1° and 152.2°, respectively. High temperature increased its hydrophobicity, and the contact angle increased to 157.1° at 200 °C. Additionally, electrochemical tests proved its better chloride ion corrosion resistance, and the corrosion potential and corrosion current of the superhydrophobic foamed concrete after 7 days were −0.190 V and 3.177 × 10^−6^ A, respectively. Therefore, the superhydrophobic bulk modification technique shows considerable potential for enhancing the durability of foamed concrete applied in various scenarios.

## 1. Introduction

Foamed concrete is a lightweight concrete characterized by high fluidity, low self-weight, reduced aggregate consumption, and excellent thermal insulation properties [1]. As an environmentally friendly and lightweight material, it significantly reduces energy consumption and has attracted wide attention worldwide in the fields of building energy conservation [2,3] and transportation construction [4].

However, several drawbacks of foamed concrete have gradually emerged during practical applications. Its inherently porous structure and the hydrophilic nature of cement-based materials facilitate the penetration of external moisture, thereby reducing its service life [5,6,7]. Furthermore, corrosive ions such as Cl^−^ and SO_4_^2−^ can infiltrate the foamed concrete through moisture as a transport medium, further causing the deterioration of the internal structure and reducing its durability [6]. Consequently, applying superhydrophobic modifications to ordinary foamed concrete to reduce its water absorption is essential for extending its service life, making them highly practical solutions.

The superhydrophobic modifications of foamed concrete can be categorized into surface and bulk modification. Both methods adhere to the “binary synergistic” principle [5,6], which involves two main steps: first, reducing the surface energy of the matrix using hydrophobic agents and, second, constructing an appropriate rough structure on the matrix surface. According to Wenzel’s theory, increased surface roughness enhances the hydrophobic effect when a material’s surface is hydrophobic (90° < CA < 150°) [8,9]. The superhydrophobic (CA > 150°) surface modification of foamed concrete primarily includes spraying and impregnation techniques. Researchers have employed various hydrophobic agents to reduce the matrix’s surface energy and utilized nanoparticles such as nano-silica (NS) and graphene oxide (GO) to construct rough structures on a specimen’s surface, so as to successfully prepare superhydrophobic-surfaced foamed concrete [7,10,11,12,13,14].

Superhydrophobic bulk modification demonstrates superior waterproofing effectiveness compared to traditional surface modifications, primarily due to its resistance to aging and peeling. Dong [15] and She [6] employed polymethylhydrosiloxane (PMHS) and isobutyltriethoxysilane (IBTS) as hydrophobic agents, respectively, successfully preparing SFC using a chemical foaming method. However, in order to address the challenges of controlling the foaming rate and mitigating the potential safety risks associated with this method, some researchers have tried to use physical foaming methods to prepare SFC. For example, Sun [5] and Xu [16] utilized polydimethylsiloxane (PDMS) to modify foamed concrete slurry, producing SFC with a contact angle exceeding 150°, thereby reducing water absorption. Shi [7] and Gao [11] produced SFC exhibiting excellent water repellency, ice resistance, and self-cleaning properties by reducing the surface energy of the matrix with silane emulsion. In addition, Chindaprasirt [17] found that calcium stearate (CS) can create a waterproof layer on the concrete capillary wall, endowing foamed concrete with remarkable hydrophobicity.

Currently, research on superhydrophobic foamed concrete (SFC) has primarily focused on the preparation and the evaluation of its superhydrophobicity, while studies on its durability are relatively limited. The durability of SFC critically impacts its service life. In addition, due to the challenges associated with preparing superhydrophobic bulk foamed concrete through physical foaming methods [16], most durability studies have concentrated on surface-modified superhydrophobic foamed concrete [10,11,12]. However, the superhydrophobic coatings on the surface of foamed concrete frequently encounter durability-related issues. They are prone to aging and peeling [5,16,18], which significantly reduce their service life during the actual application process and escalates the maintenance costs. Therefore, preparing superhydrophobic bulk foamed concrete and exploring the key factors affecting its durability are of great significance for improving the durability of foamed concrete and promoting its application and development.

This study aimed to prepare a superhydrophobic bulk foamed concrete with excellent durability using a physical foaming method and to explore the crucial factors influencing its durability. Firstly, based on the principle of “binary synergy”, polydimethylsiloxane (PDMS) was used to modify a foamed concrete slurry to ensure hydrophobicity, while calcium stearate (CS) stabilized the foam. A mesh-covered method was employed to construct micro–nano structures on the surface of the foamed concrete, enhancing its superhydrophobicity. Fourier-transform infrared spectroscopy (FT-IR) and confocal laser scanning microscopy (CLSM) were utilized to investigate the grafting status of the hydrophobic groups within the superhydrophobic foamed concrete and the construction of its surface micro–nano structures. Subsequently, various durability tests—including mechanical abrasion, high-temperature (0–200 °C) and low-temperature (−20–20 °C) resistance, ultraviolet (UV) aging, acid–alkali–saline corrosion, electrochemical tests, and outdoor exposure tests—were conducted to assess the durability performance of the superhydrophobic foamed concrete, revealing the key factors that influenced its durability. Overall, this study contributes to advancing the field of superhydrophobic bulk foamed concrete fabricated via the physical foaming method. It not only revealed the mechanism of the superhydrophobic bulk modification process but also explored the factors influencing the durability of SFC. The findings of this study can aid in the development of more sustainable functional foamed concrete for construction applications.

## 2. Raw Materials and Test Methods

### 2.1. Raw Materials

Portland cement (P·O42.5) was purchased from Conch Cement Co., Ltd., Wuhu, China, and its chemical and physical properties are listed in Table 1. Grade II fly ash (FA) was used as a supplementary cementitious material and produced by Henan Hengyuan New Materials Co., Ltd., Xinyang, China. The foaming agent was a plant-based composite foaming agent. Polydimethylsiloxane (PDMS) was a hydrophobic agent, and tetraethyl orthosilicate (TEOS) and dibutyltin dilaurate (DD) were the crosslinking agent and catalyst, respectively. Hydroxypropyl methyl cellulose (HPMC) was used for thickening and water retention, with a viscosity of 150,000 cs. Calcium stearate (CS), analytically pure, was used for foam stabilization. Polycarboxylate superplasticizer (PCE) was purchased from Subote New Materials Co., Ltd., Nanjing, China, with a 30% water-reducing rate. Wire mesh 304# was used to construct the surface micro–nano structure.

### 2.2. Mix Design

The water-to-binder ratio was 0.35, and the mix design for ordinary foamed concrete (OFC) followed the JGJ/T 341–2014 standard [5]. Additionally, 5% PDMS and 3% CS, based on the mass of the cementitious material were used. Among them, PDMS was used for the superhydrophobic modification, with TEOS serving as the crosslinking agent for PDMS and DD acting as the catalyst for the reaction. The mass ratio of the three was PDMS: TEOS: DD = 100: 10: 1. CS was used as a foam stabilizer for the foamed concrete. To improve the fluidity of the SFC slurry, 0.45% PCE was added, and to enhance thickening and water retention, 0.1% HPMC was added to the slurry. Due to hydrophobic agents usually producing a defoaming effect to some extent [6,16], more foam was incorporated during the preparation of the superhydrophobic foamed concrete to ensure that its density remained comparable to that of ordinary foamed concrete. The specific mix design is shown in Table 2.

### 2.3. Preparation of Superhydrophobic Foamed Concrete

**Figure 1:**
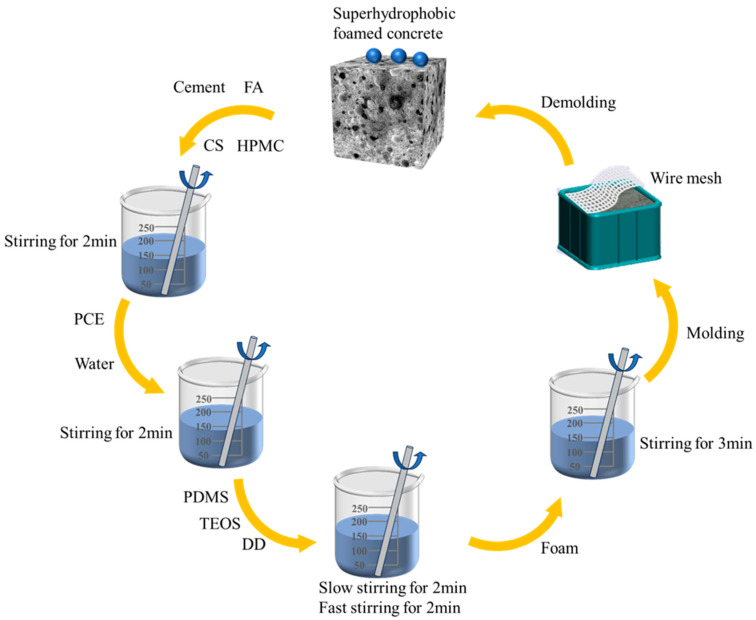
Superhydrophobic foamed concrete preparation flow chart.

Firstly, the dry materials were stirred for 2 min. Water and PCE were then added and stirred for another two minutes. Next, the hydrophobic mixture was added and stirred slowly and quickly for 2 min each until fully incorporated. Finally, we added the pre-prepared foam to the slurry, which we stirred slowly until completely dispersed. The fresh slurry was then poured into 40 mm × 40 mm × 40 mm molds covered with wire mesh at the bottom. After standing at 20 °C for 48 h, we removed the molds and placed the specimens in a curing chamber at 30 °C and 75% humidity for 7 days and 28 days for various tests. The specific preparation process is presented in Figure 1.

### 2.4. Test Methods

#### 2.4.1. Performance Test

##### Wettability

The contact angle (CA) and rolling angle (RA) of the foamed concrete surface were measured using a DSA30 contact angle analyzer from KRUSS, Berlin, Germany, at 20 °C to characterize its superhydrophobic properties. The probe liquid volumes used for measuring the contact angle and rolling angle were approximately 5 μL and 20 μL, respectively [8]. The average values from the measurements taken at no fewer than five different locations were used as the final values. The behavior of the water droplets on the specimen’s surface was recorded using a camera integrated with equipment.

##### Water Absorption and Softening Coefficient

The method for testing the volumetric water absorption rate is detailed in reference [5]. Specimens cured for 28 days were dried in an oven at 60 ± 5 °C until a constant weight before immersing in water at 20 ± 5 °C. At regular intervals, the specimens were removed, wiped with a damp cloth, and weighed to calculate the water absorption rate. Before measuring the capillary water absorption rate, the four sides adjacent to the absorption surface were sealed with epoxy resin. Once the resin had cured, tests were conducted continuously for 7 days to observe the trend in water absorption over time for each group of specimens [6]. The ratio of the compressive strength after immersion to the compressive strength before immersion was used to determine the softening coefficient of the specimens. The compressive strength test method is shown in Section Compressive Strength Test.

##### Compressive Strength Test

The compressive strength test was conducted in accordance with specification JG/T266-2011 [19]. All test blocks were dried to constant weight in a blast drying oven at 60 ± 5 °C before testing [20]. An automatic universal testing machine with a maximum range of 100 kN was used to measure the compressive strength of 40 mm × 40 mm × 40 mm cubes, with a loading rate of 1.5 kN/s.

##### Self-Cleaning

Carbon black was laid on the surface of the superhydrophobic foamed concrete, and droplets were introduced onto the specimen’s surface. The self-cleaning effect of the superhydrophobic foamed concrete was evaluated by observing the degree to which the water removed pollutants from the concrete surface [18].

##### High-Temperature Resistance Test

After measuring the initial contact angle and rolling angle of the specimen, it was placed under various high-temperature conditions for 24 h. The specimen was then removed and allowed to return to atmospheric conditions. Once the temperature stabilized to normal room temperature, the contact angle and rolling angle were measured again to evaluate the specimen’s high-temperature resistance [21].

##### Low-Temperature Resistance Test

After measuring the initial contact angle and rolling angle of the specimen, it was placed under different low-temperature conditions for 24 h. The specimen was then removed and allowed to return to atmospheric conditions. After a period of time, the contact angle and rolling angle were measured again when the temperature returned to normal room temperature. Additionally, 4 g of water droplets was placed on the surface of both OFC and SFC, which were then placed in a low-temperature chamber at −20 °C. After freezing for 24 h, the freezing condition of the specimen’s surface and the degree of deicing difficulty were observed to evaluate the low-temperature resistance of the SFC [11].

##### Ultraviolet Aging Test

Superhydrophobic foamed concrete was subjected to an ultraviolet aging chamber for accelerated aging. The UV lamp emitted a wavelength range of 315–420 nm, with an output power of 20 W. The contact angle and rolling angle were measured every 12 h over a total duration of 7 days.

##### Mechanical Wear Test

The test block was placed on 80-mesh sandpaper, with a 500 g weight positioned above it. The block was then dragged 10 cm back and forth along the horizontal direction at a constant speed as a cycle, with the contact angle measured after every five cycles. The mechanical robustness of the superhydrophobic foamed concrete was evaluated based on the change in contact angle [22].

##### Acid–Alkali–Saline Corrosion Test

Three pieces of superhydrophobic foamed concrete were selected for each group. The surfaces were ultrasonically cleaned and dried to ensure they were free of pollutants. Hydrochloric acid (HCl), sodium hydroxide (NaOH), and sodium chloride (NaCl) were, respectively, used to prepare 0.01 mol/L acid, alkali and saline solution. The specimens were soaked in different aqueous solutions for 3 days, then taken out, dried in a drying oven at 60 °C to a constant weight, and subsequently placed at room temperature to test the contact angle and rolling angle [5,21].

##### Outdoor Exposure Test

A 90-day outdoor experiment was conducted to expose the superhydrophobic foamed concrete to sunlight, rain, and the full cycle of wet and dry environmental changes in order to evaluate its hydrophobic performance.

#### 2.4.2. Microscopic Characterization

##### Confocal Laser Scanning Electron Microscopy

Confocal laser scanning microscopy (CLSM, Keyence VK-X1000, Osaka, Japan) was used to study the differences in surface roughness and 3D morphology between ordinary foamed concrete and superhydrophobic foamed concrete. The scanning area was set at 1500 µm × 1500 µm, with a scanning step of 5 µm.

##### SEM

The surface morphologies of the samples were analyzed using a scanning electron microscope (SEM, FEI Scios2 HiVac, Thermo Fisher Scientific, Wilmington, NC, USA).

##### FT-IR Test

Fourier-transform infrared spectroscopy (FT-IR, Thermo Scientific Nicolet iS20, Thermo Fisher Scientific, Waltham, MA, USA) was employed to analyze the changes in the functional groups of foamed concrete before and after superhydrophobic bulk modification.

##### Electrochemical Test

An electrochemical workstation (CHI660E, Shanghai Chenhua Co., Ltd., Shanghai, China) was used to test the potentiodynamic polarization (PP) curves of the OFC and SFC. The samples were prepared as cylinders with a radius of 2 cm and a height of 5 cm, featuring a steel bar polished with sandpaper inside, with one end connected to a wire. The foamed concrete specimens were immersed in a 3.5% NaCl solution for 7 days before the test. The foamed concrete served as the working electrode, while a platinum electrode and a saturated calomel electrode acted as the counter electrode and reference electrode, respectively. The sweep rate for the potentiodynamic polarization test was set at 1 mV/s, covering a potential range of −200 to 800 mV relative to the open circuit potential [23]. The corrosion potential and corrosion current were obtained by fitting the Tafel curve to evaluate the resistance of the superhydrophobic foamed concrete to chloride ion erosion.

## 3. Results and Discussion

### 3.1. Properties

#### 3.1.1. Wettability

**Figure 2:**
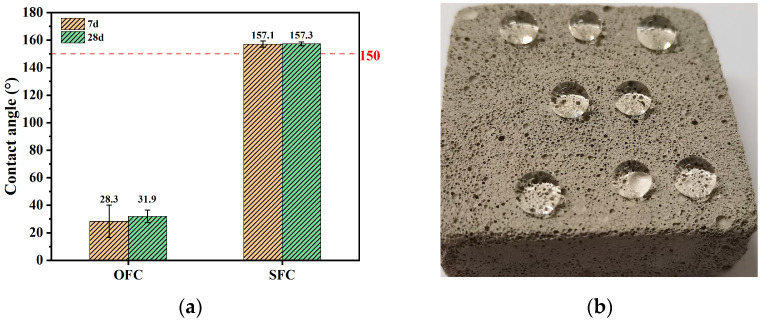
Wettability for different samples: (**a**) contact angle; (**b**) effect diagram of superhydrophobic foamed concrete.

The contact angle values of the ordinary foamed concrete (OFC) and superhydrophobic foamed concrete (SFC) are presented in Figure 2. The contact angles for the OFC at 7 days and 28 days were 28.3° and 31.9°, respectively, while those for the SFC at 7 days and 28 days were 157.1° and 157.3°, respectively. The surface contact angle of the unmodified foamed concrete was less than 90°, indicating its inherent hydrophilic characteristics. In contrast, the surface contact angle of the superhydrophobic modified foamed concrete exceeded 150° and reached a superhydrophobic state. Figure 2b shows an effect diagram of the superhydrophobic foamed concrete, where water droplets condense into balls on the surface of the material rather than spreading out to moisten the surface. The reduction in surface energy of the foamed concrete was achieved through the application of a hydrophobic agent, while micro–nano structures were constructed using wire mesh to alter the surface’s micro-morphology, forming a mastoid structure. The “air cushion effect” reduced the contact area between the droplets and the specimen surface, thereby reducing the wettability, enhancing hydrophobicity, and increasing the contact angle [13].

#### 3.1.2. Water Absorption

**Figure 3:**
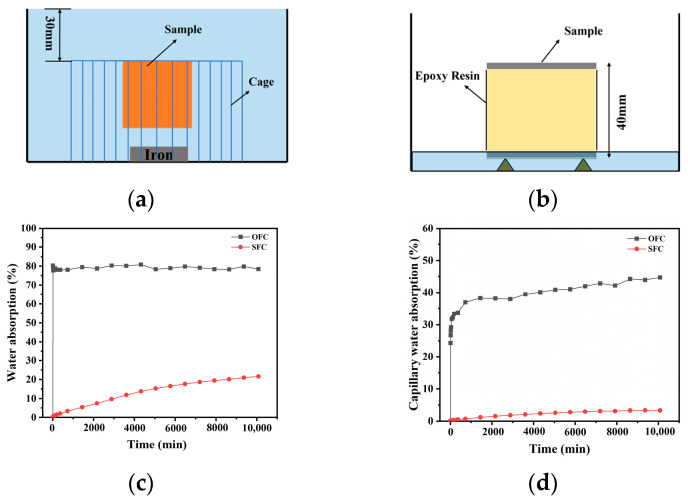
Volumetric and capillary water absorption of different samples. (**a**) Schematic diagram of volumetric water absorption; (**b**) capillary water absorption diagram; (**c**) volumetric water absorption; (**d**) capillary water absorption.

The contact angle serves as an indicator of the local hydrophobic effect of SFC, while water absorption reflects its overall hydrophobic performance. Figure 3a,b illustrate the schematic diagrams for the volumetric and capillary water absorption tests. The results of these tests for the OFC and SFC are presented in Figure 3c,d, respectively. After soaking for 7 days, the volumetric water absorption and capillary water absorption of the OFC were significantly higher, measuring 78.4% and 44.7%, respectively. However, the water absorption rates of the superhydrophobic modified foamed concrete were markedly lower, with volumetric and capillary water absorption rates of 21.6% and 3.3%, respectively, representing reductions of 72.4% and 92.6% compared to the OFC. This obvious difference demonstrates the superhydrophobic effect of SFC. The PDMS modification enhanced the hydrophobic properties of the entire matrix, leading to decreased water absorption. Additionally, the hydrophobic film formed by the hydrolysis of calcium stearate (CS) coated the surface of the cement hydration products, preventing external water from wetting the concrete and further reducing the water absorption [17].

#### 3.1.3. Self-Cleaning

**Figure 4:**
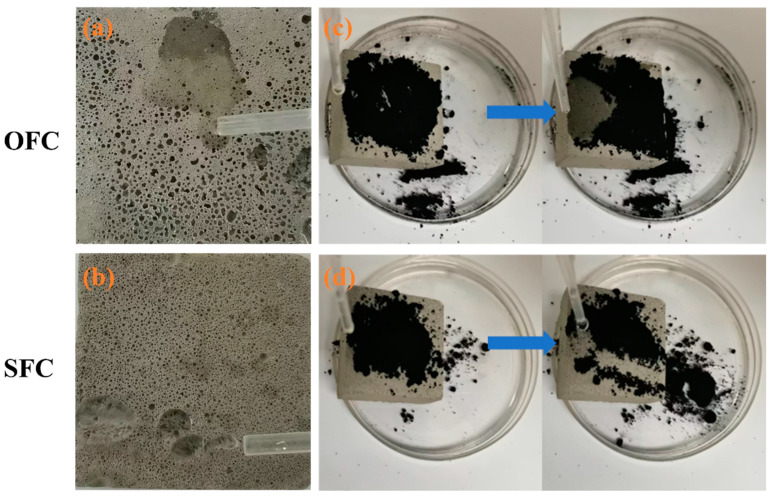
Self-cleaning diagram of SFC and OFC. (**a**) Water droplets on OFC, (**b**) water droplets on SFC, (**c**) self-cleaning test on OFC, (**d**) self-cleaning test on SFC.

The surfaces of building walls are easily polluted by particulate matter and liquid pollutants from the air, which not only detract from their appearance but also prove difficult to clean using conventional methods. Over time, this accumulation can adversely affect the aesthetics of the building. Figure 4 compares the self-cleaning effects of the OFC and SFC. Figure 4a,b show the surfaces of the OFC and SFC impacted by water flow. It can be found that water droplets were absorbed by the surface of the OFC due to its inherent hydrophilicity. In contrast, Figure 4b illustrates the bouncing reflection of water droplets on the superhydrophobic surface of SFC.

Figure 4c,d present the self-cleaning test of the OFC and SFC in air, where carbon black powder simulated particulate pollutants on the surface. When water droplets were dropped on the surface of OFC, they were quickly absorbed by the material, causing the carbon black powder to adhere stubbornly to the material. When water was dropped on the SFC surface, due to the low surface energy of the SFC surface, the water droplets immediately condensed into balls, and the carbon black powder floated on the surface of the water. When the specimen was inclined, gravity caused the droplets to roll down, carrying the particulate pollutants with them, leaving a clean surface behind. A clear trace of the rolling droplets could be seen in the middle of the specimen, demonstrating that the surface appeared clean and new. These results indicate that the SFC developed in this study not only effectively repels airborne particulate pollutants but also prevents the adhesion of liquid contaminants, showcasing excellent self-cleaning performance. This material holds significant potential for building applications, enhancing the anti-pollution capabilities and long-term maintenance of structures, positioning it as an advanced engineering material.

#### 3.1.4. Mechanical Wear Resistance

One of the significant challenges facing superhydrophobic coatings in practical applications is their inadequate mechanical wear resistance, which severely limits their widespread promotion and application. Consequently, researchers are increasingly focusing on superhydrophobic bulk modification techniques that effectively enhance wear resistance. These approaches not only improve the surface hydrophobicity of the material but also provide better mechanical stability, allowing it to maintain its excellent superhydrophobic property even after exposure to external wear.

**Figure 5:**
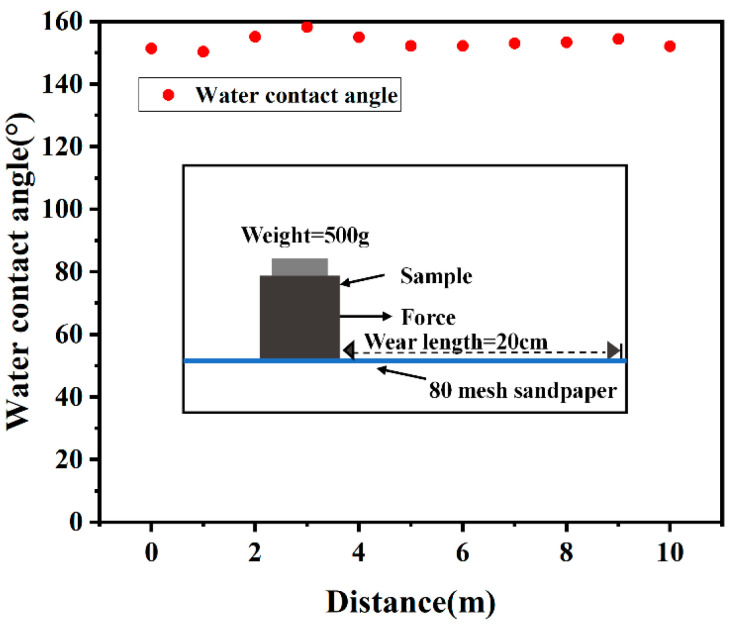
Wear resistance test.

To further explore the influence of external mechanical wear on the superhydrophobic surface of foamed concrete, a sandpaper wear experiment was designed. As illustrated in Figure 5, a 500 g weight was placed on the surface of the foamed concrete sample, which was positioned on 80-mesh sandpaper. The sample and weight were dragged back and forth horizontally over a distance of 20 cm at a constant speed for each cycle. After every five cycles, the contact angle of the specimen was measured to assess the changes in its surface hydrophobicity. The results indicated that, despite increasing the wear distance, the contact angle of the foamed concrete surface remained consistently between 150° and 160°, with minimal fluctuations. This observation demonstrates the significant advantages of superhydrophobic bulk modification regarding wear resistance.

Unlike traditional coatings, the superhydrophobic foamed concrete developed in this study retained its superhydrophobic properties even after being subjected to sandpaper wear. Remarkably, even when the mesh structure on the surface of the material was completely destroyed due to wear, the newly exposed foamed concrete surface still exhibited an excellent hydrophobic property. This phenomenon was attributed to the superhydrophobic bulk modification of the foamed concrete material, where the entire matrix is hydrophobic rather than relying solely on a surface coating. Furthermore, as the micro and nano structures on the specimen’s surface were worn down, the grinding process inherently reconstructed new micro and nano structures, preserving both the roughness of the surface and the superhydrophobic effect. In summary, through the “binary synergy” effect, under the joint modification of the surface micro–nano structure and hydrophobic substances, the foamed concrete achieved superhydrophobicity throughout its entire matrix. This superhydrophobic bulk modification is crucial to achieve excellent mechanical robustness for foamed concrete.

#### 3.1.5. Acid, Alkali, and Saline Solution Erosion

**Figure 6:**
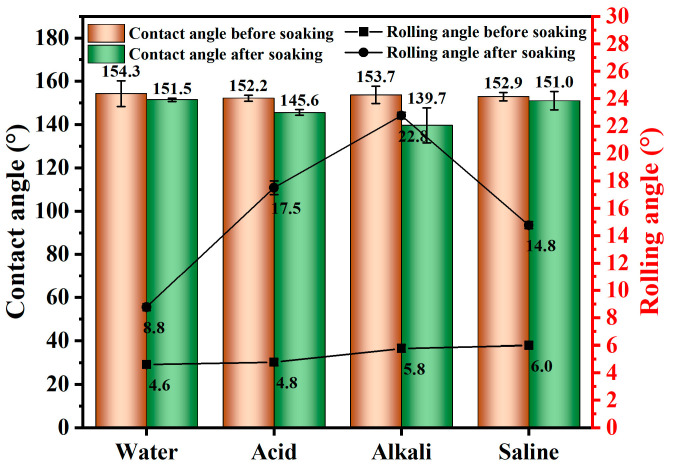
Effects of different pH values on the hydrophobicity of foamed concrete.

Foamed concrete is susceptible to corrosion from acid, alkali, and saline solutions in outdoor environments. Without adequate protective measures, such exposure can significantly degrade the performance of concrete. Therefore, studying the corrosion resistance of superhydrophobic foamed concrete is of great practical importance. Figure 6 illustrates the changes in the contact angle and rolling angle of the superhydrophobic foamed concrete after soaking in various corrosion solutions for 3 days.

After the specimen was immersed in deionized water for 3 days, the contact angle decreased from 154.3° to 151.5°, representing a reduction of 1.81%, while the rolling angle increased from 4.6° to 8.8°. Similarly, after the specimens were immersed in saline solution (PH = 7) for 3 days, the contact angle decreased from 152.9° to 151.0°, a decrease of 1.24%, while the rolling angle rose from 6° to 14.8°. After soaking in acid solution (PH = 2) for 3 days, the contact angle decreased from 152.2° to 145.6°, a decrease of 4.34%, while the rolling angle increased from 4.8° to 17.5°. For the alkali solution (PH = 12), the contact angle decreased from 153.7° to 139.7°, a reduction of 9.11%, and the rolling angle rapidly increased from 5.8° to 22.8°. These results indicate that immersion in solutions with different pH values led to varying degrees of decline in the hydrophobic properties of the SFC. Notably, the deionized water and saline solution had little influence on the superhydrophobic effect of the specimen surface, whereas the acid and alkali solutions significantly affected the degradation of this effect. Specifically, the SFC surfaces were more acid-resistant and less alkali-resistant. The deterioration of the superhydrophobic performance can be attributed to the following factors:

(1) The HCl in the acid solution reacted with calcium stearate (CS), disrupting the superhydrophobic surface structure and diminishing the hydrophobic effect of the specimen surface; (2) sodium hydroxide (NaOH) permeated the concrete through its pores, and upon drying, NaOH crystals remained on the surface, increasing the surface energy and reducing the contact angle; (3) while immersion in pure water and saline water slightly reduced the superhydrophobicity, the surfaces did not regain their original hydrophobic state after drying. Capillary action transported Ca(OH)_2_ to the surface, further increasing the surface energy and reducing the contact angle [5]. (4) The prolonged exposure of polydimethylsiloxane (PDMS) to water and chemicals led to structural densification and breakdown of its macromolecular hydrophobic chains [24]. Additionally, the water molecules adsorbing onto the PDMS chain created more polar sites, which further diminished the hydrophobic effect.

**Figure 7:**
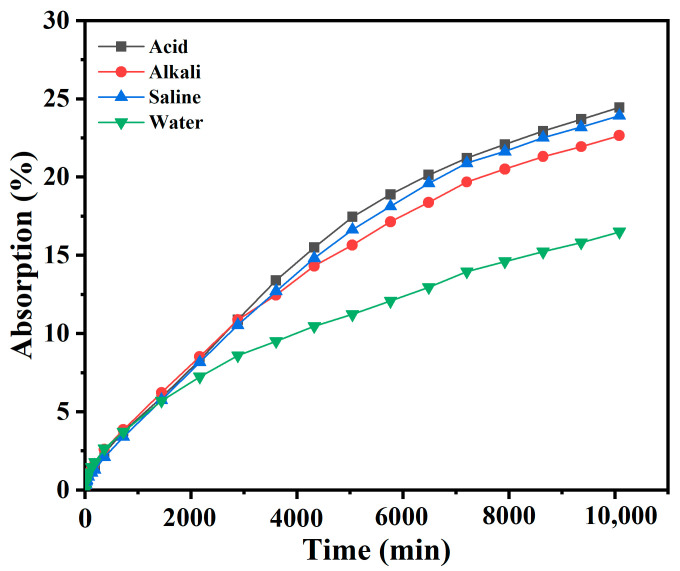
Influence of aqueous solutions with different pH values on water absorption of SFC.

External media with different pH values cause different degrees of damage to the internal structure of superhydrophobic foamed concrete. Figure 7 describes the influence of solutions with different pH on the volumetric water absorption of the superhydrophobic foamed concrete. After soaking in deionized water for 7 days, the water absorption rate was low, at 16.5%. However, the water absorption increased significantly in the acid, alkali, and saline solutions, reaching 24.5%, 22.7%, and 23.9%, respectively. As the concrete itself was alkaline, it was easily eroded in the acidic environment of the HCl aqueous solution, which led to the gradual deterioration of its performance. The Ca(OH)_2_ dissolved in an acid environment, and the internal porosity of the concrete increased, ultimately leading to an increase in water absorption. In addition to acid erosion, strong alkali corrosion also caused damage to the internal structure of the concrete [25,26]. When the aluminate in the cement clinker encountered a strong base such as NaOH, it reacted to produce sodium aluminate, which is soluble in water, which reacted with CO_2_ in the air to produce sodium carbonate, which crystallized and expanded in the capillary of the foamed concrete, causing the matrix to loosen and crack, also leading to an increase in water absorption. Similarly, chloride salts penetrated the concrete, reacting with hydration products such as Ca(OH)_2_ to form soluble chlorides. This reduced the compactness of the matrix and altered the pore structure, resulting in higher porosity and further facilitating the infiltration of chloride ions. In summary, the water absorption rate of the SFC increased through the erosion caused by the acid, alkali, and saline solutions, and the order of the degree of influence was acid > saline > alkali.

**Figure 8:**
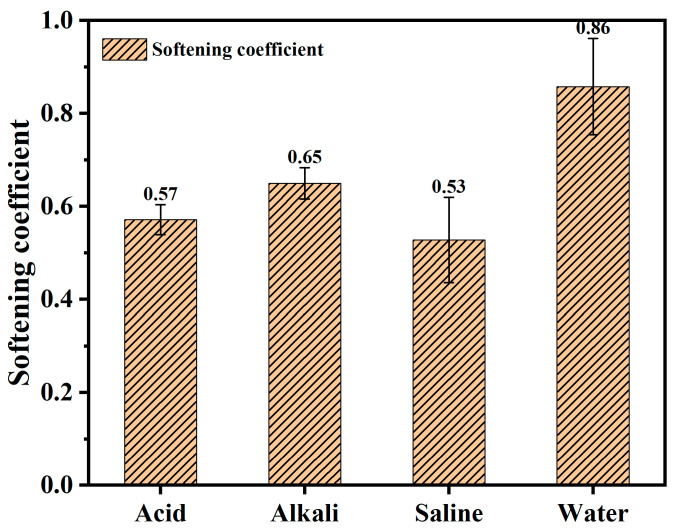
Effect of aqueous solutions with different pH values on softening coefficient of SFC.

Figure 8 illustrates the softening coefficient of the SFC after immersion in solutions with different pH values for 7 days. The trends in the softening coefficient and water absorption rate following exposure to different aqueous solutions were generally consistent. After soaking in deionized water, the specimens exhibited a softening coefficient of 0.86. However, exposure to other aqueous solutions for seven days resulted in varying degrees of reduction in the softening coefficient. Among them, the highest softening coefficient was observed in the specimens soaked in the alkaline solution with a pH of 12, followed by those immersed in an acidic solution with a pH of 2, while the lowest was recorded in specimens soaked in saline solution with a pH of 7. Strong acid corrosion and chlorine salt corrosion consumed the hydration products inside the foamed concrete, causing an increase in porosity, thus increasing the water absorption rate and reducing the softening coefficient. Overall, the exposure to acid, alkali, and saline aqueous solutions resulted in varying degrees of erosion of the superhydrophobic foamed concrete, contributing to a reduction in the softening coefficient.

#### 3.1.6. Ultraviolet Aging

**Figure 9:**
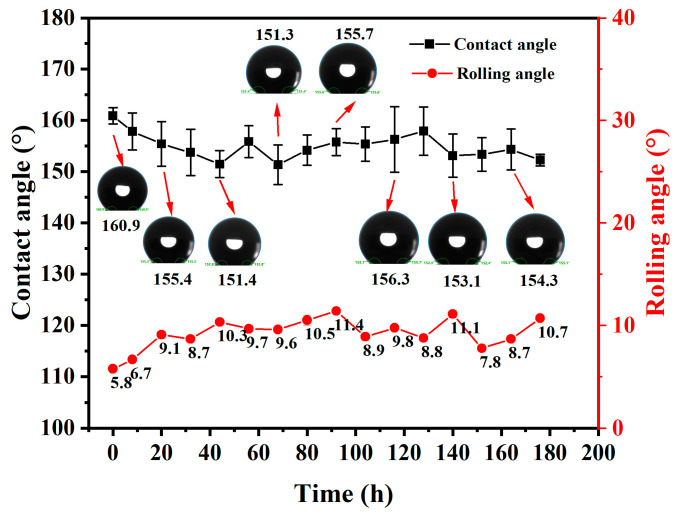
Effect of ultraviolet irradiation time on hydrophobic properties of SFC.

As a roof insulation layer, foamed concrete is subjected to significant ultraviolet radiation, which may accelerate the aging of organic waterproof substances in superhydrophobic foamed concrete, thereby diminishing its superhydrophobicity [27,28]. To evaluate the UV aging resistance of the superhydrophobic foamed concrete, an accelerated UV aging test chamber was utilized, and the results are presented in Figure 9.

After a 7-day UV aging test, the contact angle of the SFC initially decreased before stabilizing, generally showing a decreasing trend. In contrast, the rolling angle increased initially and then stabilized during the same period, exhibiting an overall upward trend. Prolonged ultraviolet irradiation led to the decomposition or oxidation of the hydrocarbon groups on the surface of the SFC, resulting in reduced hydrophobicity. For example, the Si-CH_3_ bond within the PDMS chain can break, producing volatile methane and hydrogen [29]. This indicated that UV accelerated the aging of the organic material on the surface of the SFC and reduced the hydrophobic performance. Nevertheless, the contact angle remained above 150°, and the rolling angle stabilized near 10°, indicating that the prepared SFC still exhibited and excellent hydrophobic effect and good resistance to ultraviolet aging.

#### 3.1.7. Low-Temperature Resistance

**Figure 10:**
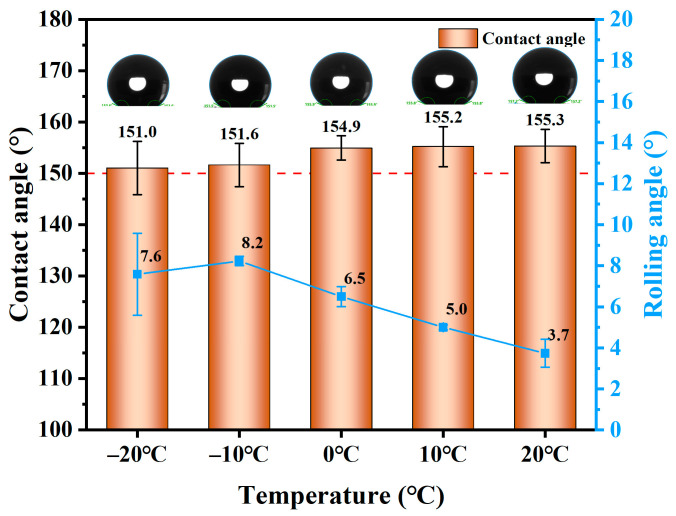
Change in hydrophobic property of SFC at low temperature.

As a building insulation material, foamed concrete is typically utilized in relatively cold environmental conditions. Therefore, studying the low-temperature resistance of superhydrophobic foamed concrete is of great significance, as it can enhance its applicability in practical projects. Figure 10 illustrates the changes in the contact angle of SFC under varying temperature conditions.

With the increase in temperature, the contact angle of the SFC surface increased gradually from 151° at −20 °C to 155.3° at 20 °C. On the contrary, the rolling angle of the SFC surface steadily decreased from 7.6° at −20 °C to 3.7° at 20 °C. At temperatures below 0 °C, the water vapor in the air condensed on the surface of the foamed concrete. As the temperature increased, the ice began to melt, moistening the surface and thereby reducing the contact angle. Additionally, at lower temperatures, water vapor liquefied on the surface, increasing the concentration of polar molecules and diminishing the hydrophobic effect. Overall, the low-temperature environment reduced the hydrophobic properties of the SFC’s surface. However, following the evaporation of water, the contact angle still remained above 150°, and the rolling angle stayed below 10°, indicating that the SFC maintained its hydrophobic state and exhibited significant low-temperature resistance.

**Figure 11:**
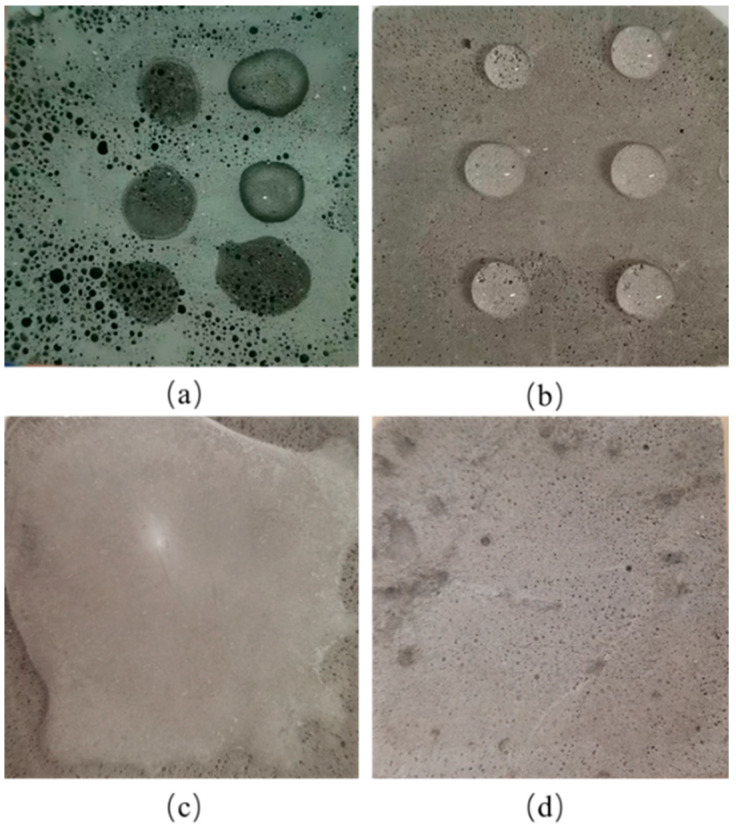
Icing resistance of different foamed concrete. (**a**) OFC’s surface, (**b**) SFC’s surface, (**c**) SFC’s surface before deicing, (**d**) SFC’s surface after deicing.

The anti-icing performance of foamed concrete at low temperatures is also important. Figure 11a shows the difference in the surface wettability between OFC and SFC at −20 °C. It demonstrates that when water droplets were placed on the surface of the OFC, they were quickly absorbed, leading to the formation of a frost layer. This indicated that the OFC at low temperature still had good wettability, which created durability problems for the specimen. Figure 11b depicts the wetting condition of the SFC surface at −20 °C. When water droplets were placed on the SFC surface, they were not absorbed but instead formed beads that turned into ice within approximately 40 s. Compared to the OFC, the SFC exhibited superior anti-icing performance. When water drops on the surface of foamed concrete, the heat transfer dynamics for the water droplets on foamed concrete involve both conduction with the surface and radiation to the surrounding air. A larger contact area between water droplets and OFC results in greater heat loss, while a smaller contact area with SFC minimizes heat loss, thus prolonging the icing time [30].

Ice repellency is also an important index for evaluating the ice resistance of superhydrophobic foamed concrete, as it reflects the adhesion between concrete and ice sheets [11]. Figure 11c,d illustrate the SFC surface before and after deicing. After 24 h of freezing, a thick layer of ice formed on the surface of the SFC; however, the ice was easily removed from the surface intact with deicing tools without damaging the surface layer of the SFC. This demonstrates that the superhydrophobic modified foamed concrete exhibited excellent anti-icing performance at low temperature, and the external water only froze to the surface of the test block but did not penetrate into foamed concrete. The adhesion between the ice sheet and the foamed concrete was minimal, facilitating a relatively straightforward deicing process.

#### 3.1.8. High-Temperature Resistance

**Figure 12:**
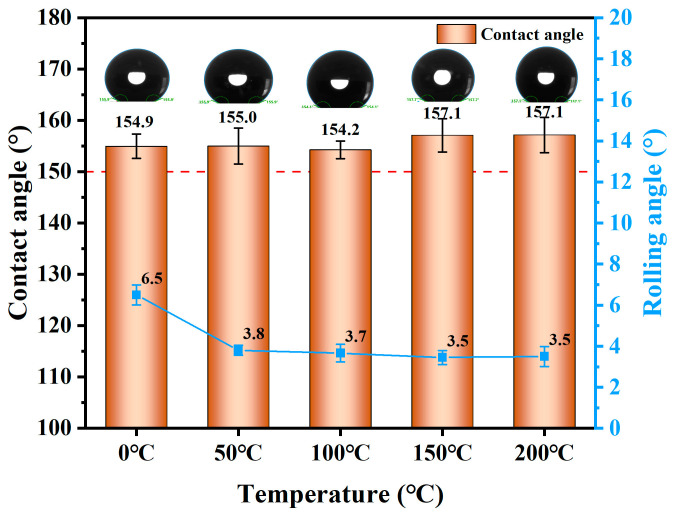
Change in hydrophobic property of SFC at high temperature.

Organic matter is prone to aging at high temperature, making it essential to study the impact of elevated temperatures on the hydrophobic performance of SFC. Figure 12 shows the influence of the heat treatment temperature on the contact angle of the SFC. The surface hydrophobicity of the foamed concrete improved gradually with increasing heat-treatment temperature, with the contact angle rising from 154.9° at 0 °C to 157.1° at 200 °C. However, the rolling angle decreased gradually at higher temperatures, from 6.5° at 0 °C to 3.5° at 200 °C. This indicates that increasing the heat-treatment temperature positively influences the hydrophobic performance of SFC. On one hand, the inorganic amorphous layer formed by the hybrid crosslinking of PDMS and TEOS had excellent heat resistance, significantly enhancing its thermal stability [21]. On the other hand, heating promoted the hydrophobic substances inside the foamed concrete to migrate from the micropores to the surface, thereby reducing the surface energy of the matrix and restoring its superhydrophobicity [10].

#### 3.1.9. Outdoor Exposure

In practical engineering applications, concrete is usually exposed to outdoor conditions, enduring prolonged exposure to wind, sunlight, rain, and other environmental factors. Therefore, in order to assess the durability of SFC in outdoor environment, this study conducted an outdoor test over a period of 90 days to evaluate the changes in its hydrophobic property.

**Figure 13:**
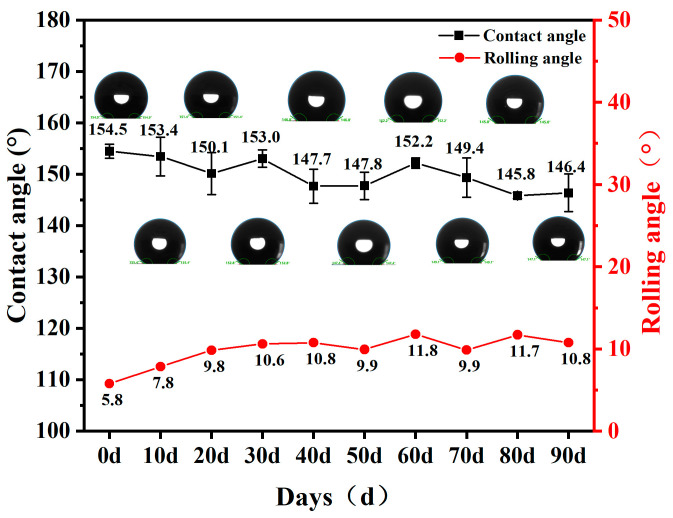
Effect of outdoor exposure test on hydrophobic property of SFC.

Figure 13 shows the change in the hydrophobic property of the SFC following a 90-day outdoor exposure test. The contact angle exhibited an overall downward trend, decreasing from an initial 154.5° to 149.4°, while the rolling angle increased from 5.8° to 9.9°. Over time, the hydrophobic effect of the specimen weakened due to the alternating dry and wet conditions experienced over the 90 days of sun and rain. Although the contact angle declined and fell below the threshold for superhydrophobicity, it remained above 140°, indicating a good hydrophobic effect. The reduction in hydrophobicity was attributed to the comprehensive effect of many external factors, with the most significant being the prolonged exposure to rain erosion and ultraviolet (UV) aging. Rain erosion caused continuous mechanical erosion of the surface of the material, and ultraviolet light accelerated the degradation of the surface’s organic material. These harsh external environments gradually weakened the superhydrophobic property of the foamed concrete. However, it is worth noting that the decrease in the contact angle was not significant, and the surface of the specimen maintained a good hydrophobic state, so as to maintain a certain degree of self-cleaning ability. This demonstrates that the SFC developed in this study can effectively withstand the challenges caused by long-term exposure to natural environments, thereby supporting its durability in outdoor engineering applications.

### 3.2. Microscopic Characterization

#### 3.2.1. CLSM Test

**Figure 14:**
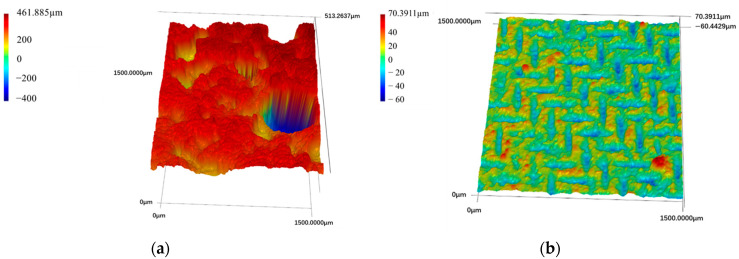
Three-dimensional topography of different foamed concrete surfaces: (**a**) ordinary foamed concrete; (**b**) superhydrophobic foamed concrete.

Figure 14 shows the 3D topography of the surfaces of the OFC and SFC. Figure 14a displays the 3D surface topography of the OFC, revealing that the surface contained pores and exhibited high surface roughness with irregular morphological changes. According to the Wenzel theory model, OFC is inherently hydrophilic, so the increase in surface roughness further improves its hydrophilic properties [9]. In contrast, Figure 14b presents the 3D topography of the SFC, covered by wire mesh and lacking pores, which reduced its surface porosity. The micro–nano structure of the wire mesh was transferred to the surface of the foamed concrete, resulting in a profile structure with periodic variations. In addition, PDMS modified the foamed concrete paste to impart hydrophobicity, reducing the surface energy of the hardened matrix and ultimately achieving a superhydrophobic surface.

#### 3.2.2. SEM Test

**Figure 15:**
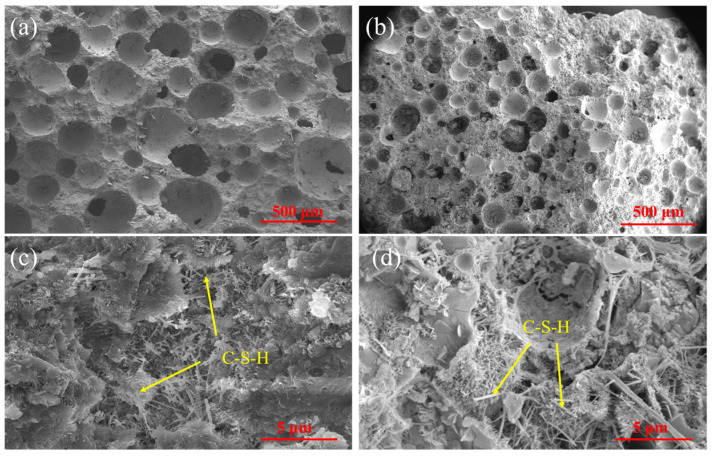
SEM images of different samples (**a**,**c**) ordinary foamed concrete (**b**,**d**) superhydrophobic foamed concrete.

The SEM images of the ordinary foamed concrete and superhydrophobic foamed concrete are shown in Figure 15. It is obvious that the internal pore diameter of the ordinary foamed concrete was larger than that of the superhydrophobic foamed concrete. CS particles were attached to the surface of the bubble and balanced the forces acting on the bubble in all directions. This exerted a remarkable bubble-stabilizing effect, resulting in relatively uniform pore size and pore distribution.

As depicted in Figure 15c, there were fibrous and foil-like C-S-H gels inside ordinary foamed concrete, and these hydration products are the main source of the compressive strength of foamed concrete. Nevertheless, these hydration products are inherently hydrophilic. As shown in Figure 15d, owing to the hydrolysis reaction of CS and the dehydration–condensation reaction of PDMS, the surface of the hydration products such as C-S-H gel was covered with a layer of hydrophobic substances, which provided an excellent waterproof effect. In addition, after superhydrophobic bulk modification, the internal morphology of the foamed concrete became rougher and conformed to the principle of “binary coordination”. Consequently, the material exhibited superhydrophobicity overall.

#### 3.2.3. FT-IR Test

**Figure 16:**
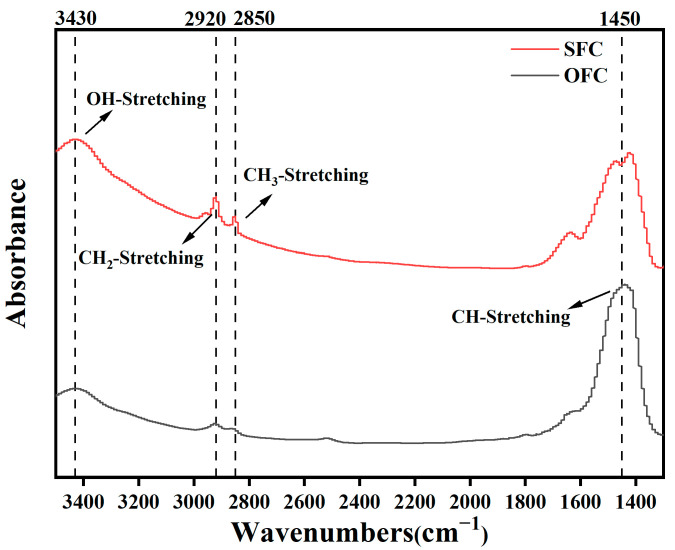
FT-IR diagrams of different samples.

As shown in Figure 16, the FT-IR spectra of the ordinary foamed concrete and superhydrophobic foamed concrete indicate that the absorption peak at 3430 cm^−1^ corresponds to the stretching vibration of the OH group [16]. The absorption peaks at 2920 cm^−1^ and 2850 cm^−1^ are attributed to the stretching vibration of CH_2_ and CH_3_ groups, respectively [6]. Additionally, the absorption peak around 1450 cm^−1^ arises from the stretching vibration of the CH group [31]. Compared to the OFC, the hydrolysis reaction induced by incorporating low-surface-energy substances such as polydimethylsiloxane (PDMS) and calcium stearate (CS) introduced additional hydrophobic groups like CH_2_ and CH_3_ into the foamed concrete. These hydrophobic groups were grafted onto the foamed concrete matrix through condensation reactions with the hydroxyl groups on the surface of the slurry, thereby reducing the surface energy of the matrix and enhancing the overall hydrophobic properties [32,33,34,35].

#### 3.2.4. Electrochemistry Test

Reinforcement bars are widely utilized in cement-based materials because of their excellent ductility and strength. The strong alkalinity (pH > 12.5) of cement-based materials typically stabilizes the passivation film on the surface of rebar. However, the presence of chloride ions in the environment can destroy the passivation film, leading to rebar corrosion [36]. The corrosion of rebar in concrete results in the formation of corrosion products that are 6–10 times the volume of the original rebar, causing cracking and spalling of the surrounding concrete, as well as pitting on the rebar due to metal dissolution. The application of superhydrophobic bulk modification has been shown to enhance the corrosion resistance of cement-based materials.

**Figure 17:**
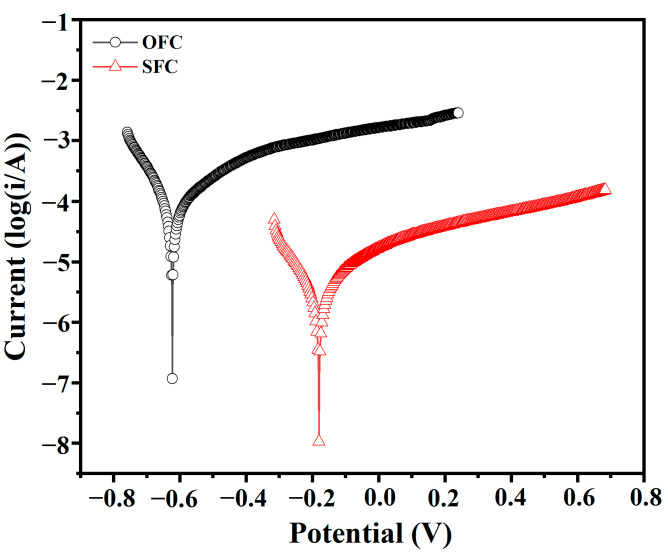
Potentiodynamic polarization curves of different foamed concretes.

Figure 17 illustrates the potentiodynamic polarization curves of the steel bars in the OFC and SFC, which are commonly employed to evaluate corrosion behavior. The corresponding corrosion potential (E_corr_) and corrosion current (I_corr_) are listed in Table 3. The corrosion potential of the reinforcement in the SFC was significantly lower, at −190 mV, compared to approximately −618 mV for the reinforcement bars in the OFC. Additionally, the reinforcement bars encapsulated in the SFC exhibited a lower corrosion current. A more positive corrosion potential and a smaller corrosion current indicate better corrosion resistance and a relatively low corrosion rate [9,37]. Therefore, compared with the SFC, reinforcement bars in the OFC experienced more severe corrosion, while the superhydrophobic bulk modified foamed concrete demonstrated effective resistance to chloride ion erosion in the external environment, thus suppressing the potential-difference-induced electrochemical corrosion between the newly exposed steel bars and the undamaged passivation film [38,39], exhibiting good corrosion resistance.

## 4. Conclusions

The inherent hydrophilicity of foamed concrete leads to durability problems. In this paper, superhydrophobic bulk foamed concrete (SFC) was prepared by a physical foaming method, and its durability was evaluated using various testing techniques. According to “binary synergy” theory, SFC was successfully prepared by using hydrophobic agents and constructing micro and nano structures, and the contact angle (CA) reached 157.3°. After soaking for 7 days, the volumetric water absorption and capillary water absorption decreased by 72.4% and 92.6%, respectively, compared with ordinary foam concrete (OFC). In addition, the dry density of the OFC and SFC was 720 kg/m^3^ and 850 kg/m^3^, respectively.

The prepared SFC had good durability. After polishing 10 m with 80-grit sandpaper and 168 h of ultraviolet radiation, the contact angles were also greater than 150°, indicating a superhydrophobic state. Aqueous solutions at different pH levels reduced the hydrophobicity of the SFC, and the alkaline substance reduced the CA by up to 9.1% after 3 d of immersion. High temperatures improved the hydrophobicity of the SFC, and the CA rose to 157.1° at 200 °C. In contrast, at a low temperature of −20 °C, the CA decreased to 151 °C. In addition, the SFC had stronger Cl^–^ corrosion resistance than the OFC, and the potentiodynamic polarization curve showed a higher corrosion potential (−0.190 V) and lower corrosion current (3.177 × 10^−6^ A).

The superhydrophobic bulk foamed concrete fabricated in this study has extensive application prospects. Firstly, the waterproofing of roofs has long been a crucial issue. SFC can maintain excellent hydrophobic performance over a long period, reducing the structural damage and maintenance costs resulting from waterproofing failures. In addition, bridges and hydraulic structures (such as dams and wharfs) are frequently exposed to water and water vapor erosion. SFC can decrease the scouring force of water flow on the surfaces of these structures, effectively prevent water infiltration, and enhance their stability and safety. SFC can also be applied in road engineering. Its internal foam structure can cushion the impact of vehicle loads to some extent, while its superhydrophobic property helps maintain the road surface’s drainage and anti-icing performance. Finally, thanks to SFC’s outstanding UV aging resistance and self-cleaning properties, it can be used on building façades to keep the building surface clean. Overall, this study expands the application scenarios of superhydrophobic foamed concrete and promotes its development.

## Figures and Tables

**Table 1:** Properties of cement (Reprinted with permission from Ref. [16]).

Specific Surface Area (m^2^/kg)	Loss on Ignition (%)	Setting Time (min)		Flexural Strength (MPa)		Compressive Strength (MPa)	
		Initial	Final	3 d	28 d	3 d	28 d
344	2.18	231	284	5.9	7.7	27.4	45.0

**Table 2:** Mix design of superhydrophobic foamed concrete.

Sample	Cement (g)	FA (g)	Water (g)	Foam (g)	CS (g)	PDMS (g)	TEOS (g)	DD (g)
OFC	675	225	315	30	0	0	0	0
SFC	675	225	315	97	27	45	4.5	0.45

**Table 3:** Corrosion potential and corrosion current of OFC and SFC.

Sample	E_corr_ (V)	I_corr_ (A)
OFC	−0.618	7.728 × 10^−5^
SFC	−0.190	3.177 × 10^−6^

## Data Availability

The raw data supporting the conclusions of this article will be made available by the authors on request.

## Abbreviations

Number	Abbreviation	Full English name
1	PDMS	Polydimethylsiloxane
2	UV	Ultraviolet
3	SFC	Superhydrophobic foamed concrete
4	OFC	Ordinary foamed concrete
5	NS	Nano-silica
6	GO	Graphene oxide
7	PMHS	Polymethylhydrosiloxane
8	IBTS	Isobutyltriethoxysilane
9	CS	Calcium stearate
10	FT-IR	Fourier-transform infrared spectroscopy
11	CLSM	Confocal laser scanning microscopy
12	FA	Fly ash
13	TEOS	Tetraethyl orthosilicate
14	DD	Dibutyltin dilaurate
15	HPMC	Hydroxypropyl methyl cellulose
16	PCE	Polycarboxylate superplasticizer
17	CA	Contact angle
18	RA	Rolling angle
19	HCl	Hydrochloric acid
20	NaOH	Sodium hydroxide
21	NaCl	Sodium chloride
22	PP	Potentiodynamic polarization
23	Ca(OH)_2_	Calcium hydroxide
24	Ecorr	Corrosion potential
25	Icorr	Corrosion current
Number	Symbol	Implication
1	CA	Contact angle
2	RA	Rolling angle
3	PH	Potential of hydrogen
4	E_corr_	Corrosion potential
5	I_corr_	Corrosion current
